# Stress, resilience, and social support among antenatal women in Jordan during the novel coronavirus pandemic: a cross-sectional study

**DOI:** 10.3389/fgwh.2025.1573789

**Published:** 2025-10-28

**Authors:** Sawsan Abuhammad, Shaher Hamaideh, Hossam Alhawatmeh, Zelal Kharaba, Karem H. Alzoubi, Heba Hijazi, Nabeel Al Yateem, Vidya Seshan, Muna Altamimi

**Affiliations:** 1 Department of Nursing, College of Health Sciences, University of Sharjah, Sharjah, United Arab Emirates; 2 Department of Maternal and Child Health, College of Nursing, Jordan University of Science and Technology, Irbid, Jordan; 3 Deparement of Nursing, College of Appliead Medical Sciences, AlMaarefa University, Dariyah, Saudi Arabia; 4 Research Center, Deanship of Scientific Research and Post-Graduate Studies, AlMaarefa University, Dariyah, Saudi Arabia; 5 Department of Adult Health, College of Nursing, Jordan University of Science and Technology, Irbid, Jordan; 6 Department of Pharmacy Practice and Pharmacotherapeutics, College of Pharmacy, University of Sharjah, Sharjah, United Arab Emirates; 7 Department of Pharmaceutical Sciences, College of Pharmacy, QU Health, Qatar University, Doha, Qatar; 8 Department of Clinical Pharmacy, Faculty of Pharmacy, Jordan University of Science and Technology, Irbid, Jordan; 9 Department of Health Care Management, College of Health Sciences, University of Sharjah, Sharjah, United Arab Emirates; 10 Department of Health Management and Policy, Faculty of Medicine, Jordan University of Science and Technology, Irbid, Jordan

**Keywords:** antenatal period, social support, stress, resilience, novel coronavirus pandemic

## Abstract

**Background and aim:**

The novel coronavirus pandemic has notably affected the psychological health of antenatal women, heightening their vulnerability to stress and raising questions about the impact of vaccination and fetal health outcomes. This study aims to examine the relationship between stress, resilience, and social support among antenatal women in Jordan during the novel coronavirus pandemic.

**Methodology:**

Using a cross-sectional approach, 434 antenatal women were surveyed in November 2021. Participants were recruited through digital platforms, including social media (Facebook and Instagram). Eligibility criteria were participants should be at least 18 years old, pregnant, living in Jordan, and proficient in English.

**Results:**

The mean perceived stress score among participants was 24.3 ± 4.4, with nearly half (49.3%) experiencing difficulty focusing, 48.9% finding daily tasks stressful, and 45.9% having trouble falling asleep. The mean social support score was 39.3 ± 9.1; the highest-rated support item was having someone available to drive them to a doctor. Pearson correlation revealed a significant positive association between resilience and social support (*r* = 0.565, *p* < 0.01). Regression analysis identified later trimester, lack of insurance, and negative life changes during the pandemic as significant predictors of higher stress among pregnant women. These findings highlight that antenatal women in Jordan experienced considerable stress and moderate social support during the pandemic, and that social support is linked to higher resilience.

**Conclusion:**

Antenatal women have experienced persistently high levels of anxiety and stress throughout the novel coronavirus pandemic. The mental health impacts are closely related to pandemic-driven factors such as isolation, interpersonal difficulties, and financial strain. Addressing these psychological outcomes and associated risk factors is essential before they worsen and impact both mothers and their unborn children.

## Introduction

The novel coronavirus pandemic, marked by loss and isolation, has created major psychological stress for many individuals (1–3). During crises like the pandemic, daily life is heavily disrupted by factors such as school shutdowns, limited contact with family and friends, enforced isolation, altered routines, and heightened health risks (4–6). In difficult situations, providing mental health support is a critical component of both healthcare delivery and community-based disaster response, especially for those most impacted (1, 7).

Recent research indicates that although pregnant women generally face lower physical risks from the novel coronavirus pandemic compared with others, their perceived stress has increased—particularly concerning the safety of vaccination and its potential effects on fetal health (8, 9). Stress experienced during pregnancy can increase susceptibility to postpartum anxiety and depression, sometimes leading to more severe mood disorders (10). Several studies have reported that both pregnant women and new mothers had experienced higher stress and diminished psychosocial wellbeing during the early stages of the pandemic (11, 12). Meta-analyses also confirm that rates of depression and stress had been elevated among women during the pandemic compared with prepandemic levels (13). Globally, there have been frequent reports of heightened stress-related symptoms among antenatal women throughout the period of the pandemic (14).

Slight elevations in prenatal stress can result in postnatal anxiety and depression symptoms, even among healthy or low-risk women. Preventive strategies are more effective when symptoms are identified early (15). Moreover, maternal stress and anxiety during the antenatal period are linked to a wide range of potential developmental, behavioral, and health outcomes for children (16). These effects can include difficulties with attention, regulation of stress, and temperament, extending into childhood (17).

One of the most significant protective elements for antenatal women is perceived social support, which can reduce the risk of negative psychological outcomes postpartum. People commonly use coping strategies to manage stress during pandemics such as the novel coronavirus pandemic. Regardless of whether individuals seek support from others, coping strategies are a core part of stress management after crises (18, 19).

According to Vella and Pai (20), resilience is a continuous process that involves adaptive responses in the face of severe adversity. Traits such as optimism and perseverance are key to resilience (21). However, as Fletcher and Sarkar (22) note, theoretical frameworks for resilience remain incomplete. Some research has shown that resilience acts as a mediator between stress and psychological symptoms, although its moderating effect is less clear. For instance, Ma et al. (18) describe resilience as both a mediator and a moderator in the relationship between anxiety and stress among antenatal women. Meanwhile, Anyan and Hjemdal (19) found that resilience mediated the relationship with some psychological problems but found no moderating influence on anxiety symptoms.

Lazarus and Folkman (23) define coping as ongoing cognitive and behavioral efforts to manage internal and external stressors, often influenced by personality traits (21). During the novel coronavirus pandemic, coping strategies included seeking health-related information or avoiding exposure to distressing content. Many healthcare centers in Jordan reduced their hours or closed, limiting access to support and thereby affecting mental health (6).

Protective resources like social support and positive coping are crucial for navigating the mental health challenges presented by the coronavirus pandemic, particularly among young people and students (4, 24). In Jordan, Abuhammad et al. (1) found that social support from families diminished during lockdowns, contributing to increased emotional distress among women. Pandemic containment measures inadvertently reduced the social networks available to antenatal women, resulting in higher stress. Prior research has demonstrated that perceived social support benefits antenatal women both in the short and in the long term (25). Large-scale studies have linked higher perceived social support to lower levels of anxiety and stress. Despite evidence that social support can reduce stress and increase resilience, there is a lack of research specifically examining how these variables interact among antenatal women in Jordan during pandemic conditions (1). Most existing studies have focused on Western or high-income countries, leaving a gap in understanding how these dynamics play out in middle eastern contexts with different cultural, social, and healthcare structures. Given the unique social fabric and potential vulnerabilities in Jordan, particularly with disruptions caused by the pandemic, it is essential to investigate these relationships to inform locally relevant interventions (25). Therefore, this study aims to examine the relationship between stress, resilience, and social support among antenatal women in Jordan during the novel coronavirus pandemic, addressing this critical gap in the literature and supporting the design of targeted mental health programs in the Jordanian context.

## Method

### Design

A cross-sectional correlational design was used in this study. This cross-sectional design measures exposures and outcomes at a single time point; temporal ordering cannot be established, and therefore, only associations—not causal effects—can be inferred.

### Sample and sampling

In this study, antenatal women were recruited using convenience sampling in November 2021. Invitations were distributed through social media, professional organizations, and referrals within Jordan. Eligible participants were required to be Jordanian, pregnant, at least 18 years old, and fluent in either Arabic or English. The survey was available in both languages: the English version matched the quality of this manuscript, and the Arabic version was professionally translated and back-translated to ensure accuracy and consistency. To ensure comprehension and minimize language barriers, participants could choose their preferred language. A total of 500 invitations were sent to achieve a minimum sample size of 400, based on an anticipated small effect size, a significance level of 0.05, and a statistical power of 0.95. For language equivalence, the authors employed forward translation, expert panel reconciliation, back-translation, and cognitive pretesting in a Jordanian pilot prior to full launch, ensuring semantic and conceptual equivalence across Arabic and English versions.

Participants were asked screening questions at the start of the survey to confirm their nationality and current residence in Jordan. However, as with many online surveys, the authors relied on self-reported residency and could not independently verify the physical locations of the participants. This is noted as a potential limitation of our recruitment approach.

### Instrument

#### Perceived stress scale

The Perceived Stress Scale (PSS) was used to measure the perception of stress. The PSS measures the level to which a person's life situation is considered stressful (26). Items in the PSS were created to identify how overwhelming, impulsive, and overburdened respondents find their lives. The scale consists of direct questions about the level of stress and individual experiences. A higher score will suggest a high level of perceived stress, and a lower score will suggest a low level of perceived stress (26). The authors administered the 10-item PSS in Arabic/English. Although the PSS-10 has no prescriptive diagnostic cutoffs, the authors report commonly used interpretive bands (0–13 low, 14–26 moderate, and 27–40 high) alongside continuous scores and interpret categories cautiously. The Cronbach alpha was 0.92.

#### Brief resilient coping scale

This study will use the Brief Resilient Coping Scale (BRCS, 27) to measure resilience and coping tendencies by capturing tendencies to cope with stress adaptively. It measures the tendency to cope with stress adaptively by using problem-solving strategies even in challenging situations. Furthermore, the scale uses a five-point scale response that ranges from 1 (does not describe me at all) to 5 (describes me very well). When added, the sum score ranges from 4 to 20. The BRCS was administered in Arabic using a forward–back translation with expert review. The authors present conventional bands (4–13 low, 14–16 medium, and 17–20 high) and additionally analyze the BRCS as a continuous variable to avoid loss of information.

#### Emotional/informational/tangible support

The MOS Social Support Survey (28) will be used in this study to assess psychological, informative, and actual support. The Medical Outcomes Study (MOS) is designed by RAND. The patient-designed survey assesses the accessibility of help across a range of areas. The MOS Social Support Survey is a self-administrated, multifaceted, 19-item test designed to assess individuals with common and curable long-term conditions on the support systems they have access to. Four sections of the survey have been validated: pleasant social contacts, emotional and informative guidance, affectionate support, and tangible assistance. The 19-item MOS-SSS was used in Arabic. Because universal cutoffs are not established, the authors report the total score (range 19–95) and domain means (1–5). In sensitivity analyses, the authors also categorize support by tertiles for descriptive purposes.

### Data analysis

SPSS version 28 was used to analyze the data. The priori level of significance of 0.05 was used, and all tests were two-sided. Beyond descriptive and correlational analyses, the authors fit multivariable linear regression models predicting PSS-10 scores from resilience (BRCS) and social support (MOS-SSS), adjusting for age, parity, trimester, education, income, living area (urban/rural), insurance, smoking status, news exposure, and pandemic-related life changes. To probe effect modification, interaction terms were tested (e.g., Social Support × Living Area; BRCS × Parity). Model assumptions (linearity, homoscedasticity, and multicollinearity) were assessed and robust standard errors were used. As part of sensitivity analyses, the authors examined ordinal models using PSS-10 categories.

## Results

### Demographic variables

The response rate was high 434 (89%). More than half of the participants were from a low-income family background (see Table 1).

**Table 1:** Demographic characteristics of antenatal women during the COVID-19 pandemic (*N* = 434).

Variable	Category	*n*	%
Insurance	No	147	33.6
	Yes	287	65.5
Income (USD)	<400	272	62.1
	400–600	148	33.8
	600–800	12	2.7
Education	Primary/secondary	144	32.9
	Diploma	94	21.5
	University student	28	6.4
	Bachelor's	146	33.3
	Postgraduate	22	5.0
Previous pregnancies	0	120	27.4
	1	130	29.7
	2	110	25.1
	≥3	74	16.9
Evaluation of life status	Excellent	26	5.9
	Very good	77	17.6
	Good	90	20.5
	Accepted	137	31.3
	Not good	101	23.1
Pregnancy fetus	One	335	76.5
	Two	90	20.5
	Three	9	2.1
COVID-19 test	No	212	48.4
	Yes, positive	122	27.9
	Yes, negative	81	18.5
	Yes, awaiting result	19	4.3
Life change after challenge	Much better now	21	4.8
	Somewhat better now	96	21.9
	Unchanged	120	27.4
	Somewhat worse	106	24.2
	Much worse	91	20.8
Trimester (GW)	First	129	29.5
	Second	182	41.6
	Third	118	26.9
Living area	City	342	78.1
	Village	91	20.8
Smoking	No	292	66.7
	Yes	141	32.2
Challenging circumstance vaccination	No	162	37.0
	Yes	271	61.9
Frequency of news exposure	Never	55	12.6
	Barely	120	27.4
	Sometimes	163	37.2
	Always	95	21.7
History of flu shot	No	150	34.2
	Yes	170	38.8
	Maybe	113	25.8

### Description of stress among pregnant mothers in the novel coronavirus pandemic era

The results showed that pregnant mothers experienced an average stress level of 24.3 ± 4.4. “I had difficulty focusing on what I was working on”, 215 (49.3), “whatever you did felt stressful to me”, 209 (48.9), and “I had trouble falling asleep”, 197 (45.9) were the responses that received the highest level of agreement (see Table 2).

**Table 2:** Description of stress among antenatal women during the COVID-19 pandemic.

Item	Rarely *n* (%)	Sometimes *n* (%)	All times *n* (%)
Unable to control important things in life	208 (48.0)	164 (37.9)	61 (14.1)
Confident in the ability to deal with own problems (first)	154 (35.6)	173 (40.0)	106 (24.5)
Confident in the ability to deal with own problems (second)	148 (34.3)	147 (34.0)	137 (31.7)
Things going as wanted	146 (33.9)	167 (38.7)	118 (27.4)
Difficulties accumulating, unable to overcome	190 (43.9)	140 (32.3)	103 (23.8)

### Description of support among pregnant mothers in the novel coronavirus pandemic era

The mean level of assistance of pregnant mothers was 39.3 ± 9.1. How much does someone who can drive you to the doctor when you need them is one of the things with the highest score. The other scores are as follows: those who understand your difficulties, 190 (43.8%), and someone you trust or with whom you can discuss yourself or your concerns, 196 (45.2%), and 206 (47.5%) (see Table 3).

**Table 3:** Description of social support among antenatal women during challenging circumstances.

Item	Never *n* (%)	Sometimes *n* (%)	Always *n* (%)
Help with needs during bed rest	83 (19.1)	163 (37.6)	188 (43.3)
Person to take to the doctor	68 (15.7)	160 (36.9)	206 (47.5)
Someone to prepare meals	97 (22.4)	158 (36.4)	179 (41.2)
Help with daily routine when sick	97 (22.4)	151 (34.8)	186 (42.9)
Someone to listen when you need to talk	102 (23.5)	153 (35.3)	179 (41.2)
Someone to give good advice during crisis	93 (21.4)	151 (34.8)	190 (43.8)
Someone to provide information about the situation	94 (21.7)	166 (38.2)	174 (40.1)
Someone you trust/talk about yourself	78 (18.0)	160 (36.9)	196 (45.2)
Someone you need advice from	98 (22.6)	149 (34.3)	187 (43.1)
Someone to share worries/fears with	89 (20.5)	170 (39.2)	175 (40.3)
Someone to suggest how to deal with problems	85 (19.6)	155 (35.7)	194 (44.7)
Someone who understands your problems	88 (20.3)	156 (35.9)	190 (43.8)

### Correlation between stress, resilience, and social support among antenatal women during the novel coronavirus pandemic

Pearson's analysis demonstrated a significant association between stress, resilience, and social support (*r*^2^ = −.581, *p* = .001). In particular, greater social support was related to higher resilience (see Table 4).

**Table 4:** Correlations between stress, resilience, and social support among antenatal women during the COVID-19 pandemic.

Item	Stress	Resilience	Social support
Stress	1	−0.026	−0.003
Resilience	−0.026	1	0.565**
Social support	−0.003	0.565**	1

** *p* < 0.01 (two-tailed) for correlations. *N* = 434.

### Predictors of stress among antenatal women during the novel coronavirus pandemic

The regression model was significant (*F* = 4.6, *p* = .001). Predictors of increased stress included being in a later trimester (*B* = .165, *p* = .001), lacking insurance (*B* = .122, *p* = .006), experiencing major life changes during the pandemic (*B* = −.256, *p* < .001), and feeling pressure (*B* = .120, *p* = .018). These results indicate that antenatal women in higher trimesters, without insurance, or under additional pressure, had higher stress levels (see Table 5).

**Table 5:** Predictors of stress among antenatal women during the COVID-19 pandemic.

Predictor	B	Std. error	Beta	*t*	*p*-Value
(Constant)	15.394	1.595	—	9.651	0.000
Trimester of baby	0.736	0.200	0.165	3.678	0.000
Living area	−0.589	0.365	−0.071	−1.611	0.108
News exposure frequency	−0.214	0.154	−0.061	−1.390	0.165
Smoking status	−0.742	0.198	−0.170	−3.755	0.200
Age	0.020	0.034	0.029	0.569	0.570
Insurance	−0.869	0.316	−0.122	−2.749	0.006
Income	−0.020	0.269	−0.004	−0.076	0.940
Pregnancy months	0.086	0.165	0.027	0.524	0.601
Life status	−0.114	0.141	−0.041	−0.809	0.419
Education level	0.313	0.319	0.044	0.981	0.327
Resilience level	−0.051	0.142	−0.120	−0.362	0.018
Life change during challenge	−0.755	0.158	−0.265	−4.762	0.000
Social support	0.084	0.051	0.075	1.639	0.102

Dependent variable: Stress.

## Discussion

This study confirms that antenatal women in Jordan experienced higher stress levels and symptoms of psychological distress during the novel coronavirus pandemic. Different pandemic-related challenges—such as economic pressures, social isolation, relationship struggles, and health fears—were each uniquely linked to stress. These results underscore the importance of mitigating stress in antenatal women to protect their mental health during public health crises.

Since the outbreak of the novel coronavirus pandemic in December 2019, various studies have documented the psychological toll resulting from the pandemic, with some researchers forecasting compounded effects for antenatal women (29, 30). Multiple sources demonstrate heightened stress and mental health issues for expectant mothers. For example, Woody et al. (31) found that approximately 7%–12% of antenatal women report significant stress and mental health conditions during the antenatal period. Some studies observed that stress and depression scores among antenatal women were higher during the novel coronavirus pandemic compared with previous years (32). Early data from the pandemic also reported increased clinical stress among pregnant individuals (33, 34). While methodological differences make exact comparisons difficult, our findings reveal an even greater proportion of clinically significant depression compared with research conducted in the initial months of the pandemic.

The present study also found that perceived support was low among antenatal women during the coronavirus pandemic. In Jordan, extended family networks, evolving family structures with urbanization, and patterns of healthcare access during the pandemic likely shaped perceived support and stress. These contextual features should inform interpretation and the tailoring of interventions (1). Stress levels ranging from 25 to 37 have been reported elsewhere, which is notable, since this research was conducted during a state of emergency in Ontario, Canada (12, 33). As the coronavirus pandemic continued and social distancing remained in place, rates of depression and stress may have increased further (35). Our sample also reported high stress; 30–40 participants across several stress scale items identified major concerns about the antenatal period and health. Consistent with meta-analytic findings, our results show higher stress and anxiety among antenatal women during the novel coronavirus pandemic compared with previous years (36). The sample expressed greater worry about factors such as their partner's presence at delivery and their infant's health, compared with prepandemic samples. Global syntheses consistently report elevated perinatal depression and anxiety during the pandemic. A rapid review and meta-analysis estimated antenatal depression at ∼25.6% and anxiety at ∼30%–31% (7). Addressing these specific concerns is essential for effective public health and mental health intervention programs for antenatal women. In addition, increased risks of the pandemic and relationship issues were linked to higher stress, anxiety, and insomnia. Social distancing increased anxiety and stress but was not associated with sleep problems. Financial strain was tied to both stress and insomnia (37). Together, these outcomes show that the indirect consequences of the pandemic collectively worsen mental health challenges.

In this study, the only significant relationship found was between resilience and social support, with increased support linked to greater resilience. Previous research supports that significant stress events like disasters and pandemics can increase psychological distress (38, 39), and stress during the antenatal period can affect child development (39). Social support is widely regarded as a buffer against mental health challenges (40). This study found that negative perceptions about the pandemic and the degree of social support both relate to mental health symptom severity. One surprising finding was that women reporting high social support also had higher stress scores compared with those with moderate support; this may reflect that women experiencing greater stress are more likely to seek or perceive higher levels of support. It is also possible that in the context of acute crises, perceived support increases in response to escalating stress rather than serving solely as a buffer. This nuanced relationship highlights the complexity of psychosocial responses during the antenatal period and crisis situations, as echoed in other studies from the region (25). Importantly, the overall trend in the literature remains that social support generally mitigates stress and enhances resilience among pregnant women. Those with limited support and negative views about the coronavirus pandemic experienced the worst mental health outcomes, while robust social support lessened these effects. When negative cognitive assessments were low, social support had less impact on mental health severity (41). These findings suggest social support is especially protective against mental health problems when pandemic-related stress is high (42, 43).

Our research identified later trimester, lack of insurance, and increased pressure as predictors of higher stress during the novel coronavirus pandemic. Previous studies also report that as stress and anxiety rise, relationship satisfaction may decline, and postpartum depression can be linked to marital difficulties (44, 45).

In terms of maternal and child health, psychological stress during the antenatal period can have both immediate and lasting effects (46). For example, Dancause et al. (47) associate stress exposure during the antenatal period with higher risks of miscarriage and premature birth. Turcotte-Tremblay et al. (48) also suggest that maternal psychological health impacts the physical and mental development of offspring (47). Therefore, public health programs should prioritize mental health support for antenatal women, especially concerning pandemic-specific challenges like social distancing and relationship strain. Targeted interventions are vital to reduce mental health burdens and support healthy development in children.

### Limitations

This study has many limitations, first, cross-sectional design measured exposures and outcomes simultaneously, and therefore, temporal ordering is unknown and causal inference is not supported. All constructs were assessed via self-report questionnaires, which are subject to recall and social-desirability biases and do not replace clinical evaluation. The authors did not conduct clinician-administered diagnostic interviews for anxiety or depression; accordingly, the results reflect symptom severity rather than diagnoses. Finally, online convenience sampling and self-reported residency may introduce selection and misclassification biases, and unmeasured confounding and reverse causation remain possible.

### Implications of the study

The results of this study highlight several clinical and policy implications. Foremost, they indicate the need for increased focus on supporting the mental health of expectant mothers, especially given the persistent stress and anxiety months into the novel coronavirus pandemic. The findings affirm that mental health outcomes are tied to the indirect effects of the pandemic—such as isolation, relationship conflict, and financial hardship. Proactive measures to address psychological stress and its risk factors are crucial to prevent further harm to mothers and babies (49). As a resolution to help address mental health issues among antenatal impacted women, integrating digital mental health services into healthcare systems should include training healthcare providers on the importance of maintaining the confidentiality of maternal mental health data (50), particularly in countries like Jordan, could help reduce negative outcomes associated with pandemic stress (51).

## Conclusion

In summary, this cross-sectional survey of antenatal women participants in Jordan reported moderate perceived stress and moderate social support. Bivariate analyses indicated little to no direct correlation between stress and either resilience or social support, whereas resilience and social support strongly and positively underscored the fact that women who seek or perceive greater support also tend to report higher resilient coping. Moreover, multivariable models suggested that later gestational trimester, lack of health insurance, and pandemic-related life changes/pressures were associated with higher stress, highlighting structural and situational factors that may co-occur with psychological burden during pregnancy. Taken together, the study signals a sustained mental health need among pregnant women during public health crises.

## Data Availability

The raw data supporting the conclusions of this article will be made available by the authors without undue reservation.
